# Spontaneous tumor lysis syndrome (STLS) during biopsy for burkitt lymphoma: a case report

**DOI:** 10.1186/s12887-024-04679-1

**Published:** 2024-03-23

**Authors:** Sirui Pan, Qiyang Shen, Jianfeng Zhou, Tao Li

**Affiliations:** 1 Department of Oncology, Children’s Hospital of Nanjing Medical University, Nanjing, China; 2 Department of Pediatric Surgery, Children’s Hospital of Nanjing Medical University, Nanjing, China

**Keywords:** Spontaneous tumor lysis syndrome, Biopsy, Hyperkalemia, CRRT, Burkitt lymphoma, Pediatric

## Abstract

**Background:**

Tumor lysis syndrome (TLS) is a hematologic oncological emergency characterized by metabolic and electrolyte imbalances. On breakdown of tumor cells, enormous amounts of potassium, phosphate, and nucleic acids are released into systemic circulation. TLS mainly occurs during chemotherapy. However, there are rare incidences of spontaneous tumor lysis syndrome (STLS) prior to commencement of therapy.

**Case presentation:**

In the case being reported, the child had just undergone a biopsy. As the incision was being closed, there was a sudden onset of high fever, arrhythmia, severe hyperkalemia, hypocalcemia, and acidosis. Following timely symptomatic treatment and continuous renal replacement therapy(CRRT), the child’s laboratory results improved, and organ function was restored to normal. The final pathological diagnosis confirmed Burkitt lymphoma. The boy is currently on maintenance chemotherapy.

**Conclusions:**

TLS is a potentially life-threatening complication in hematologic oncology. Several important conclusions can be drawn from this case, reminding clinicians to: (1) be fully aware of the risk factors of TLS and evaluate the level of risk; (2) pay attention to the possibility of STLS during operation, if surgical procedures are necessary and operate with minimal trauma and in the shortest time possibly; (3) take preoperative prophylaxis actively for high-risk TLS patients, including aggressive fluid management and rational use of diuretics and uric-acid-lowering drugs. In addition, this case confirms the effectiveness of CRRT for severe STLS.

## Introduction

TLS is a hematological tumor emergency. It is caused by rapid tumor cell lysis, which results in the release of metabolites into the systemic circulation and is mostly caused by chemotherapy. The resultant imbalances can lead to potentially fatal metabolic abnormalities and are mainly manifested as hyperkalemia, hyperphosphatemia, hyperuricemia, and hypocalcemia [1]. These metabolic disorders increase the risk of serious complications, such as acute kidney injury(AKI), arrhythmias, convulsions, and even death [2]. STLS, however, is particularly rare, especially when occurs intraoperatively. This article reports a case of STLS that took place during a biopsy operation performed on a child with Burkitt lymphoma, in the hope of raising awareness and vigilance of the disease among clinicians.

## Case report

### Patient information

**Fig. 1:**
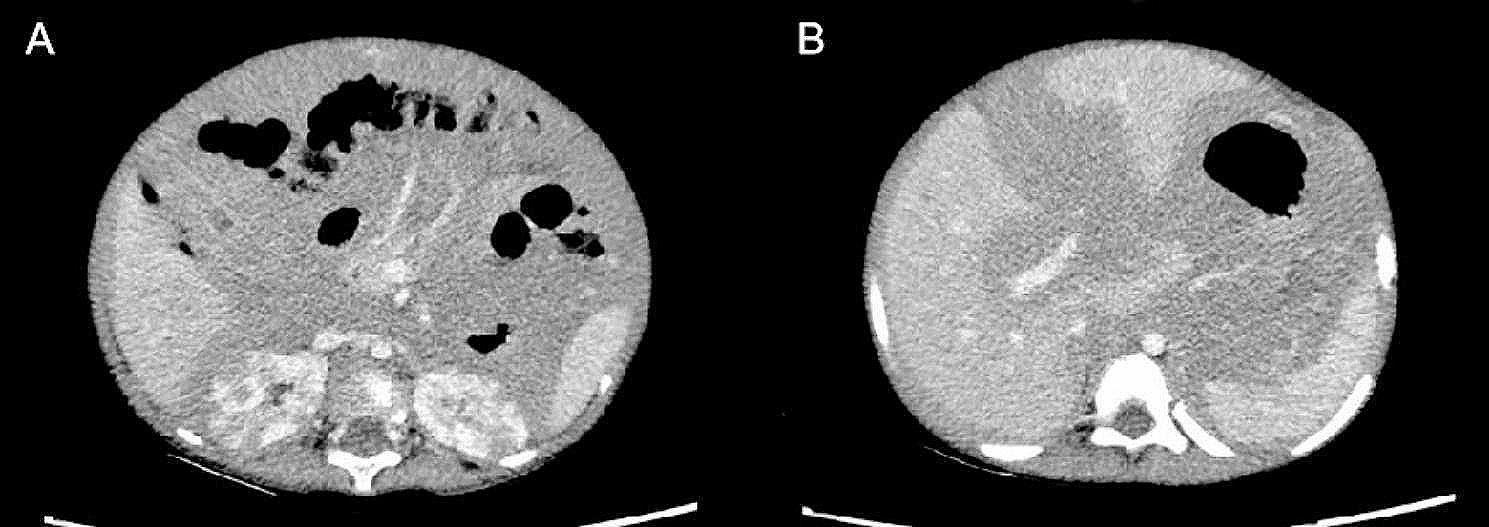
(**A**) The kidneys showed multiple hypodense shadows, the intestinal wall was significantly thickened, and the intestinal space was not clearly developed. (**B**) The liver was enlarged with uneven density. Diffuse hypodense shadows were seen around the spleen and in the abdominal cavity

A two-year-old boy was admitted to hospital with distension for one day.He was sweaty and had dense papules scattered on his head and face. The distension was immediately apparent, with an abdominal circumference of 59 cm and visible venous shadowing in the abdominal wall. A huge mass could be palpated during digital rectal examination. Computer Tomography (CT) revealed diffuse lesions on the liver, gallbladder, kidneys, intestinal tubes, and peritoneum as well as neoplastic lesions (indicative of lymphoma), left oblique inguinal hernia and bilateral pleural effusions (Fig. 1). The abdominal ultrasound revealed multiple hypoechoic masses in the abdomen-pelvic cavity; likewise in the liver; multiple segments of thickened intestinal wall (considering lymphoma). The results from an electrocardiogram were normal. However, serum biochemistry tests were abnormal, showing aspartate aminotransferase of 137U/L, lactate dehydrogenase (LDH) of 1698U/L, and α-hydroxybutyrate dehydrogenase of 1132U/L. Serum electrolytes were close to normal levels. Uric acid level was 902umol/L. Urinalysis yielded excess of urobilinogen, ketone, protein and specific gravity of 1 0.035. Sternal bone marrow puncture showed no obvious abnormalities. Bone marrow biopsy was not performed.

### Therapeutic intervention

Due to the high tumor burden, intravenous hyperhydration (1500ml/m^2^), alkalization of urine (sodium bicarbonate 1.5 g/d) and diuresis (furosemide 10 mg/d) were administered preoperatively. The average daily urine output was 415 ml and uric acid decreased slightly to 877umol/L two days later.

### Event process

The tumor was shown to be exceptionally large, and the child’s abdominal distension progressed rapidly. In order to confirm the diagnosis as soon as possible, an open tumor biopsy was performed on the third day of admission. General anesthesia was induced using intravenous mivacurium chloride and maintained by sevoflurane via endotracheal tube. After locating the tumor by B-ultrasound, a 3 cm wide transverse incision was made into the upper abdomen. The tumor was seen at the lower margin of the right lobe of the liver and was adherent to surrounding tissues. Omentum was thickened, edemateous and was pale in color. One cubic cm samples of both the tumor and omentum tissue were removed and sent in for pathological examination. Rapid pathology revealed that the tumor was malignant and had metastasized to the omentum.

The child suddenly developed tachycardia (∼ 190 beats/min) as the abdominal incision was being closed. Electrocardiography revealed widened QRS complexes and high-pointed T waves. Both physical cooling and dexibuprofen embolization were performed, as his body temperature had risen to 41.3 °C. In addition, an intravenous lidocaine injection was administered, and the child’s heart rate gradually decreasing to 25 beats/min. This prompted chest compression and an intravenous injection of epinephrine, after which the heart rate converted to a sinus rhythm of ∼ 95 beats/min. A blood sample sent for laboratory examination revealed a serum potassium level of 6.99 mmol/l; total calcium of 1.09 mmol/l: PH of 7.041; BE of -14.1. Despite multiple intravenous injections of sodium bicarbonate, calcium gluconate, epinephrine, insulin and furosemide, the patient’s heart rate remained unstable. A diagnosis of STLS was considered.

After a temporary return to normal heart rhythm and autonomous respiration, the patient was immediately transferred to the surgical intensive care unit (SICU). The initial blood results in the SICU showed that potassium levels had risen to 7.38 mmol/l and blood uric acid to 1145 umol/l, while total calcium concentration had fallen to 1.18 mmol/l. Alanine transaminase (ALT) was recorded at 1909 U/L and oxaloacetic transaminase (AST) at 7306 U/L. CRRT was initiated due to persistently elevated serum potassium levels. A continuous venous-venous hemodiafiltration (CVVHDF) mode was adopted. After the initiation of CVVHDF, the arrhythmias decreased in frequency and after 6 h, serum potassium had dropped to 4.92 mmol/l. In addition, blood uric acid decreased to 207 umol/l at 4 days postopderatively. The final pathological diagnosis confirmed Burkitt lymphoma.

### Outcomes

**Fig. 2:**
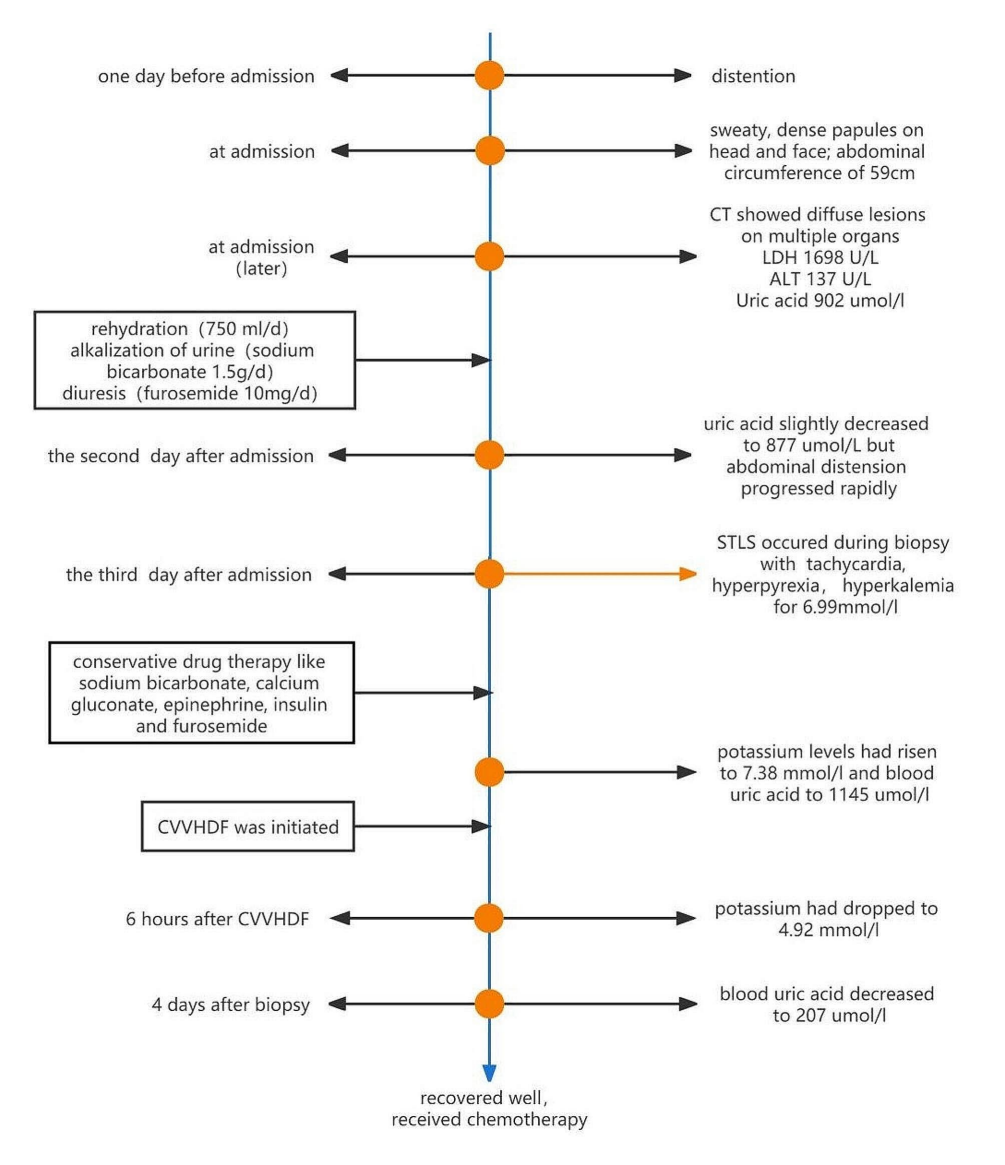
The clinical timeline of the patient

The patient was weaned from CVVHDF 4 days post-operation and was successfully extubated six days after the operation. His liver gradually recovered, exhibiting stable bloodwork results(ALT of 248 U/L, AST of 127 U/L, alkaline phosphatase (ALP) of 119 U/L, γ-glutamyl transferase (GGT) of 142 U/L). We observed that kidney function returned to normal over the next 10 days. The abdominal circumference was reduced to 55 cm. Prior to chemotherapy, rasburicase, which can reduce uric acid, hydration and alkalinization were administered to prevent TLS. He is currently on maintenance chemotherapy and has had no further episodes of TLS(Fig. 2).

## Discussion

Existing literature suggests that STLS is a very rare condition. In this case, the child experienced sudden onset of STLS towards the end of a biopsy, which was complicated by severe hyperkalemia and arrhythmia. Timely intervention using CVVHDF saved the patient’s life and restored function to organs damaged during this episode of STLS.

First reported by Cohen et al. in 1980, TLS is a life-threatening oncological emergency experienced by patients with hematological tumors [3]. TLS usually occurs 48–72 h after the start of chemotherapy and is most common in patients with acute lymphoblastic leukemia (ALL) and non-Hodgkin lymphoma (NHL) [4, 5]. STLS occurs occasionally, with an incidence of about 1.08% of hematological malignancy patients [6]. Reported triggers include corticosteroid therapy, radiotherapy, anesthesia and fever [7–11]. However, cases similar to ours, where STLS manifested during an operation but was successfully treated, are extremely rare worldwide.

The rapid lysis of tumor cells lead to the release of massive quantities of intracellular contents into the bloodstream, including anions, cations, proteins and nucleic acids. The release and subsequent catabolism of nucleic acids can result in hyperuricemia. In this case, crystals precipitate in the renal tubules as uric acid concentrations increase, which may lead to renal insufficiency or failure [12].

According to Cairo-Bishop’s definition of TLS in 2004, the laboratory criteria include: i) ≥ 2 abnormal serum values at presentation and ii) at minimum 25% increase or decrease in calcium, uric acid, potassium, or phosphorus levels either three days prior to the start of chemotherapy or within seven days following chemotherapy commencement [13]. The diagnostic criteria for clinical TLS are the same as laboratory TLS with one or more clinical complications, including renal insufficiency (serum creatinine ≥ 1.5 times the upper limit of normal), arrhythmias, seizures, or sudden death. In our case, the initial examination failed to diagnose laboratory TLS. However, when the patient suddenly developed arrhythmias, severe hyperkalemia, and hypocalcemia with preceding hyperuricemia during the biopsy operation, this met the clinical criteria of TLS diagnosis.

The mortality rate of TLS in hematological malignancies and solid tumors is 21.4% and 33%, respectively [5]. Thus, prevention, or at least early identification, of TLS is crucial. In 2010, the International TLS Expert Group formulated recommendations to define the potential risk of TLS from solid tumors and hematologic malignancies in children and adults. They defined three levels of risk based on the probability of TLS occurrence: low (1%); medium (1–5%) and high risk (5%)^[14]^. Risk factors for TLS in tumor patients usually include disease types; tumor characteristics (high tumor load, rapid tumor growth, significant sensitivity to chemotherapy) and elevated baseline lactate dehydrogenase or uric acid levels [5, 14–17]. In our case study: (1) pre-operative imaging evidence showed that the tumor had multi-organ infiltration and biochemical LDH was significantly raised, indicating a high tumor burden; (2) Abnormal metabolic functions resulted in high fever during operation, which can induce TLS; (3) The patient had hyperuricemia, in which uric acid deposition caused renal injury; (4) Mivacurium chloride, a non-depolarizing muscle relaxant, was used during the operation, as it has been suggested that depolarizing muscle relaxants can cause hyperkalemia [7, 11]. (5) The surgical biopsy of the tumor resulted in the destruction of tumor cells, prompting tumor lysis; (6) Further research into existing literature revealed that most intraoperative STLS occurred during abdominal closure, suggesting that changes in abdominal pressure could be a predisposing factor for TLS.

With regards to TLS, the adage “prevention is better than a cure” holds true. Hyperuricemia is one of the major risk factors associated with TLS, as it can cause AKI, which in turn increases the risk of mortality [2, 18]. Allopurinol is recommended for the prevention of low to medium-risk TLS, whereas urate oxidase is the preferred urate-lowering agent in high-risk patients [16, 17]. Furthermore, adequate hydration can improve intravascular volume; enhance renal perfusion and glomerular filtration; and further promote the excretion of uric acid, potassium and phosphorus, all reducing the risk of TLS. In this case, the tumor load was high, so prophylactic hydration was performed before surgery. However, the fluid infusion volume was still insufficient and the uric acid level was poorly controlled. Aggressive hydration is defined as excessive hydration with 2.5 (maximum 3) l/m2 of intravenous crystalloid per day to achieve a target urine volume of at least 4 ml/ (kg/h) [17].

Once TLS occurs, it is crucial to correct electrolyte abnormalities and administer CRRT if necessory [15]. Indications for CRRT in TLS patients are similar in patients with other causes of AKI, such as significant fluid overload; uremia; and severe electrolyte and metabolic disorders [5, 6, 19]. In this case, the patient had severe hyperkalemia during his biopsy operation, resulting in arrhythmia. Unfortunately, although the child rapidly received corrective treatments (including the injection of calcium gluconate, insulin, sodium bicarbonate), the outcome was poor. Given the poor effect associated with conservative treatment of hyperkalemia, hypocalcemia and acidosis, we opted to perform CVVHDF. Fortunately, this timely intervention caused the potassium level to decreased rapidly( 6 h after the start of CRRT) and resulted in restored kidney function following treatment. Thus, when severe hyperkalemia (potassium level ≥ 7 mmol/l) occurs in TLS, timely CRRT can potentially produce a good prognosis [17].

## Conclusion

TLS is a potentially life-threatening complication, particularly if it occurs during the treatment of childhood malignancies and particularly those that are lymphoproliferative. Clinicians should be fully aware of the risk factors of TLS and use them to determine the level of risk for each TLS patient with malignant tumors. Such a strategy can aid in early identification, leading to timely treatment and avoidance of death caused by TLS. Clinicians need to pay attention to the possibility of STLS during operation, if surgical procedures are necessary. Patients with high risk of TLS need active pre-operative prophylaxis, including aggressive fluid management and rational use of diuretics and uric-acid-lowering drugs. Furthermore, in the event of severe TLS, prompt CRRT should be considered as a viable option to save the patient.

## Data Availability

All data generated or analysed during this study are included in this published article.

## Abbreviations

TLS: Tumor lysis syndrome
STLS: Spontaneous tumor lysis syndrome
CRRT: Continuous renal replacement therapy
AKI: Acute kidney injury
CT: Computer tomography
LDH: Lactate dehydrogenase
SICU: Surgical intensive care unit
ALT: Alanine transaminase
AST: Oxaloacetic transaminase
CVVHDF: Continuous venous-venous hemodiafiltration
ALP: Alkaline phosphatase
GGT: γ-glutamyl transferase
ALL: Acute lymphoblastic leukemia
NHL: Non-Hodgkin lymphoma
